# Diagnostic performance of contrast‐enhanced mammography for suspicious findings in dense breasts: A systematic review and meta‐analysis

**DOI:** 10.1002/cam4.7128

**Published:** 2024-04-25

**Authors:** Shu‐ting Lin, Hong‐jiang Li, Yi‐zhong Li, Qian‐qian Chen, Jia‐yi Ye, Shu Lin, Si‐qing Cai, Jian‐guo Sun

**Affiliations:** ^1^ Department of Radiology The Second Affiliated Hospital of Fujian Medical University Quanzhou Fujian China; ^2^ Department of Bone The Second Affiliated Hospital of Fujian Medical University Quanzhou Fujian China; ^3^ Center of Neurological and Metabolic Research The Second Affiliated Hospital of Fujian Medical University Quanzhou Fujian China; ^4^ Department of Neuroendocrinology, Group of Neuroendocrinology Garvan Institute of Medical Research Sydney New South Wales Australia; ^5^ Department of Urinary Surgery The Second Affiliated Hospital of Fujian Medical University Quanzhou Fujian China

**Keywords:** contrast‐enhanced mammography, dense breasts, meta‐analysis

## Abstract

**Purpose:**

Contrast‐enhanced spectral imaging (CEM) is a new mammography technique, but its diagnostic value in dense breasts is still inconclusive. We did a systematic review and meta‐analysis of studies evaluating the diagnostic performance of CEM for suspicious findings in dense breasts.

**Materials and Methods:**

The PubMed, Embase, and Cochrane Library databases were searched systematically until August 6, 2023. Prospective and retrospective studies were included to evaluate the diagnostic performance of CEM for suspicious findings in dense breasts. The QUADAS‐2 tool was used to evaluate the quality and risk of bias of the included studies. STATA V.16.0 and Review Manager V.5.3 were used to meta‐analyze the included studies.

**Results:**

A total of 10 studies (827 patients, 958 lesions) were included. These 10 studies reported the diagnostic performance of CEM for the workup of suspicious lesions in patients with dense breasts. The summary sensitivity and summary specificity were 0.95 (95% CI, 0.92–0.97) and 0.81 (95% CI, 0.70–0.89), respectively. Enhanced lesions, circumscribed margins, and malignancy were statistically correlated. The relative malignancy OR value of the enhanced lesions was 28.11 (95% CI, 6.84–115.48). The relative malignancy OR value of circumscribed margins was 0.17 (95% CI, 0.07–0.45).

**Conclusion:**

CEM has high diagnostic performance in the workup of suspicious findings in dense breasts, and when lesions are enhanced and have irregular margins, they are often malignant.

## 1 INTRODUCTION

According to data released by the International Agency for Research on Cancer, new cases of breast cancer surpassed those of lung cancer for the first time in 2020, reaching 2.26 million cases.^1^ High breast density is considered an independent risk factor for breast cancer. Women with high fibroglandular content were shown to have a 4.6 times higher risk of breast cancer than the average risk.^2^ More than 50% of women under 50 years of age have been reported to have dense breasts.^3^ Superimposition of the dense glands has been shown to reduce the diagnostic performance of unenhanced breast imaging modalities, such as mammography.^4^ Conversely, the diagnostic performance of contrast‐enhanced breast magnetic resonance imaging (MRI) is far less affected by breast density.^5^ However, its use is limited due by high costs, poor visualization of suspicious calcifications, and many contraindications.

Contrast‐enhanced spectral imaging (CEM) is a novel breast imaging technique that combines contrast‐enhanced and digital mammography (DM) techniques. Compared to contrast‐enhanced breast MRI, CEM is less expensive, has a shorter duration, is more comfortable, and provides similar anatomical and functional information on breast lesions.^6^ Besides traditional mammography, MRI is currently considered to be the most sensitive examination method for the diagnosis of breast cancer.^7^ Pötsch et al.^8^ compared the diagnostic sensitivity and specificity of CEM and contrast‐enhanced MRI (CE‐MRI) for breast cancer. It was found that the sensitivity of CE‐MRI was higher than that of CEM, and the specificity was lower than that of CEM. High background enhancement (BPE) may be one of the reasons for the reduced specificity of MRI, while CEM has been shown to have a lower BPE than that of MRI.^6^ Moreover, CEM has a high spatial resolution with approximately 10 times the lesion detail of breast MRI.^9^ In addition to the diagnostic performance, CEM is also popular in patients in clinical applications, due to its lower time and cost, and higher comfort. A study^10^ compared patients' preferences for the two examination methods, and the results showed that patients had a significantly higher preference for CEM and a significantly lower anxiety rate. Therefore, CEM had good diagnostic and clinical application prospects for the diagnosis of suspected lesions in dense breasts.

Therefore, we aimed to conduct a systematic review and meta‐analysis of the diagnostic performance of CEM in the diagnostic workup of suspicious lesions in patients with dense breasts and of the differences in CEM presentation between benign and malignant lesions in dense breasts.

## 2 METHODS AND MATERIALS

### 2.1 Search strategy and selection criteria

We registered our systematic review and meta‐analysis on PROSPERO (CRD42022337160). This systematic review was conducted and reported according to the Preferred Reporting Items for Systematic Review and Meta‐Analysis (PRISMA) 2020 guidelines.

To investigate the performance of CEM in the diagnostic workup of suspicious lesions in patients with dense breasts, the literature published in the PubMed, Embase, and Cochrane databases was searched. The search terms included "dense," "density," “contrast‐enhanced spectrum mammography,” “spectral contrast‐enhanced mammography,” “contrast enhancement spectrum mammogram,” “contrast enhanced digital mammography,” “mammary glands, human,” and related free words. The specific search strategies for each database are provided in the supplementary material (Additional File S1). The search took place on August 6, 2023, with no start time limit.

The inclusion criteria were (1) subjects with breast density American College of Radiology (ACR) category of C or D, (2) all the patients underwent CEM, and (3) sufficient raw data was to be extracted directly or indirectly, including true‐positive (TP), true‐negative (TN), false‐positive (FP), and false‐negative (FN) results.

The exclusion criteria were (1) screening studies of CEM (2) non‐English literature, and (3) studies with incomplete data, duplicate articles, case reports, animal and cell studies, reviews, and other irrelevant studies.

### 2.2 Data extraction and quality assessment

The authors extracted the data from each study independently, using 2 × 2 tables and by contacting the authors if data were not available in the text. Two authors extracted the data from each included study, and when disagreements were encountered, agreement was reached via discussions with the third author. The following information was extracted from each study: author, year of publication, patient age, study design, sample size, number of lesions, number of benign and malignant lesions, baseline examination of suspicious lesions, and the numbers of TP and FN results, or TN and FP results. These data are shown in Table 1.

**TABLE 1:** Characteristics of the included studies.

Study	Year	Age	Study design	Patient no.	Lesion no.	Benign no.	Malignant no.	Baseline screening	TP	FP	FN	TN
Ainakulova et al.^11^	2021	24–78	Retrospective	151	155	66	89	Mammography, ultrasound	85	7	4	59
Anwar et al.^12^	2021	29–72	Prospective	32	40	9	31	Mammography	28	6	3	3
Azzam et al.^13^	2020	27–69	Prospective	37	63	27	36	Mammography	32	3	4	24
Chalabi et al.^14^	2021	NR	Prospective	74	74	24	50	Mammography	49	5	1	19
Cheung et al.^15^	2014	22–69	Retrospective	89	100	28	72	NR	70	6	2	22
Lu et al.^16^	2020	15–75	Prospective	115	131	67	64	Clinical symptoms	60	8	4	59
Mohamed et al.^17^	2021	21–66	Prospective	25	25	11	14	Clinical symptoms	14	4	0	7
Mokhtar et al.^18^	2014	20–70	Prospective	60	60	16	44	Mammography, ultrasound	43	8	1	8
Qin et al.^19^	2020	NR	Retrospective	114	144	110	34	Mammography, ultrasound, MRI	28	4	6	106
Sudhir et al.^20^	2021	24–72	Prospective	130	166	79	87	Clinical symptoms	84	15	3	64

Abbreviations: FN, false‐negative; FP, false‐positive; MRI, magnetic resonance imaging; NR, not recorded; TN, true‐negative; TP, true‐positive.

The QUADAS‐2 was used to evaluate the quality of the included studies, investigating risk of biasin studies in key areas such as patient selection, index test, reference standard, and flow and timing. The clinical applicability of the first three areas was also analyzed. Each question has a “yes,” “no,” or “unclear” answer option. “Yes” indicated a low risk of bias or high applicability. “No” indicated a high risk of bias or low applicability. “Unclear” indicated that sufficient data were not extracted from the literature to make a judgment. The quality assessments were cross‐checked independently by two authors. Disagreements were resolved via group discussions. Finally, the quality evaluations were recorded in Review Manager V.5.3.

### 2.3 Statistical analysis

The analysis was performed using Stata V.16.0 and Review Manager V.5.3 software. Hierarchical bivariate models were used to calculate summary sensitivity, summary specificity, summary positive likelihood ratio (PLR), summary negative likelihood ratio (NLR), and summary diagnostic odds ratio (DOR), all with their respective 95% confidence intervals (CI). The area under the curve (AUC) was calculated using a summary receiver operating characteristic (SROC) model. The secondary outcome was the association of CEM enhancement and margins with subsequent malignancy, and ultimately the OR value was used to indicate the ratio of benign and malignant outcomes for the corresponding lesion characteristics. The results of enhancement and margin analysis were obtained by the following sub‐analyses. The first sub‐analysis considered the presence of lesion enhancement (homogeneous, heterogeneous, or rim enhancement) at CEM and its association with subsequent malignancy at the reference standard. The second sub‐analysis considered the presence of lesion margins (circumscribed or non‐circumscribed) at CEM and its association with subsequent malignancy at the reference standard. We used the chi‐squared test and bivariate I‐squared statistic to assess the heterogeneity across the studies. If bivariate *I* ^2^ > 50%, heterogeneity existed, and the source of heterogeneity was further analyzed. First, we plotted a SROC curve to assess whether there was a threshold effect. When the results of the included studies were distributed as “shoulder arm” points on the SROC curve plane, there was a threshold effect among the included studies. In addition, a meta‐regression, subgroup analysis, and sensitivity (article‐by‐article exclusion) analysis were used to evaluate the sources of the heterogeneity.

## 3 RESULTS

### 3.1 Study selection

**FIGURE 1:**
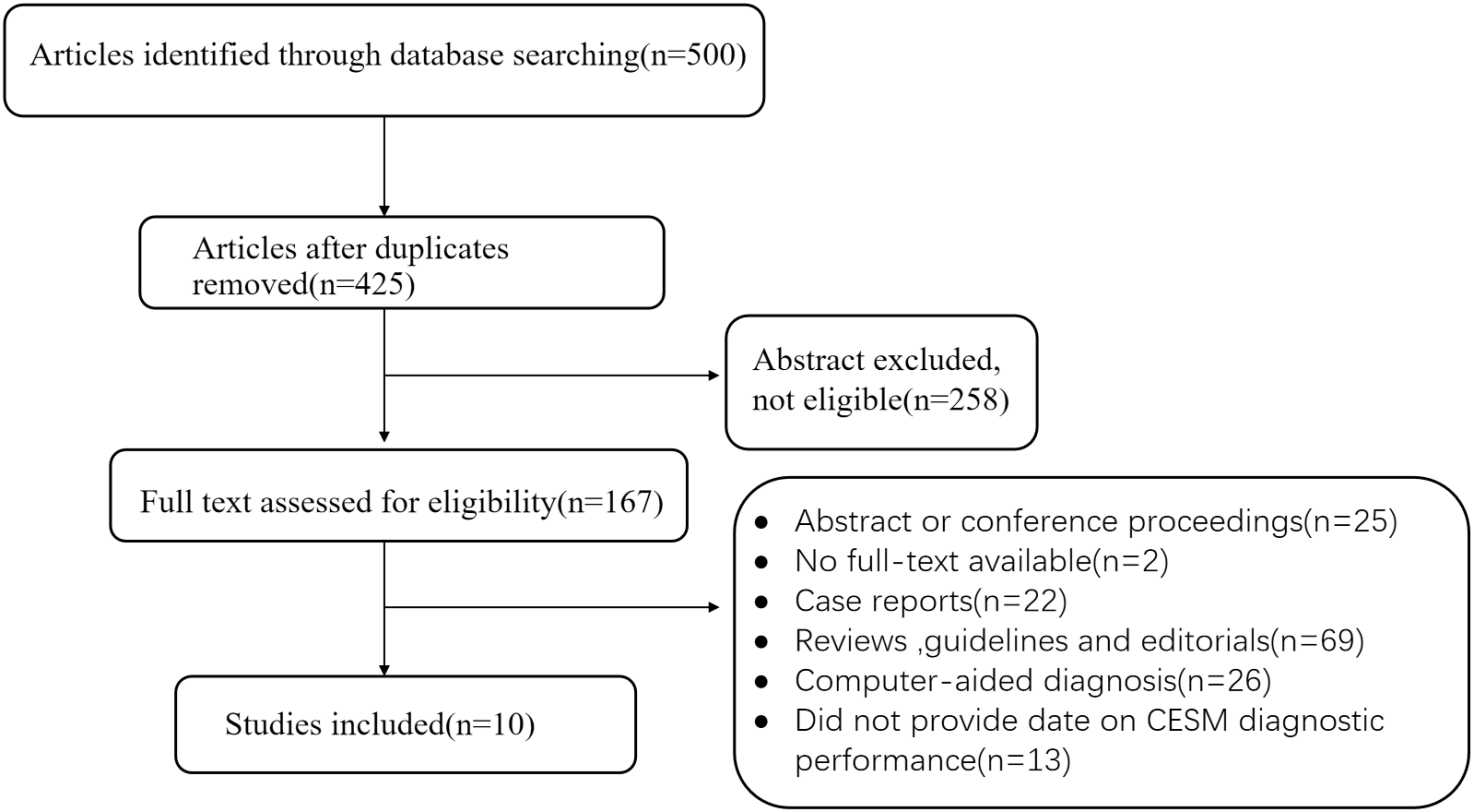
Flowchart of literature search and screening.

A total of 367 studies were retrieved by searching the PubMed, Embase, and Cochrane databases, and 22 duplicate studies were excluded. The titles and abstracts of the remaining 345 articles were read and 194 irrelevant articles were excluded. After reading the full texts of the remaining articles, 141 reports were removed. Ten studies were included in this meta‐study, which included 827 patients. These 10 studies reported the diagnostic performance of CEM for the workup of suspicious lesions in patients with dense breasts. Seven of these studies also reported information on enhancement in benign and malignant lesions. Figure 1 shows the flowchart of the literature search and screening.

### 3.2 Characteristics and quality of studies

**FIGURE 2:**
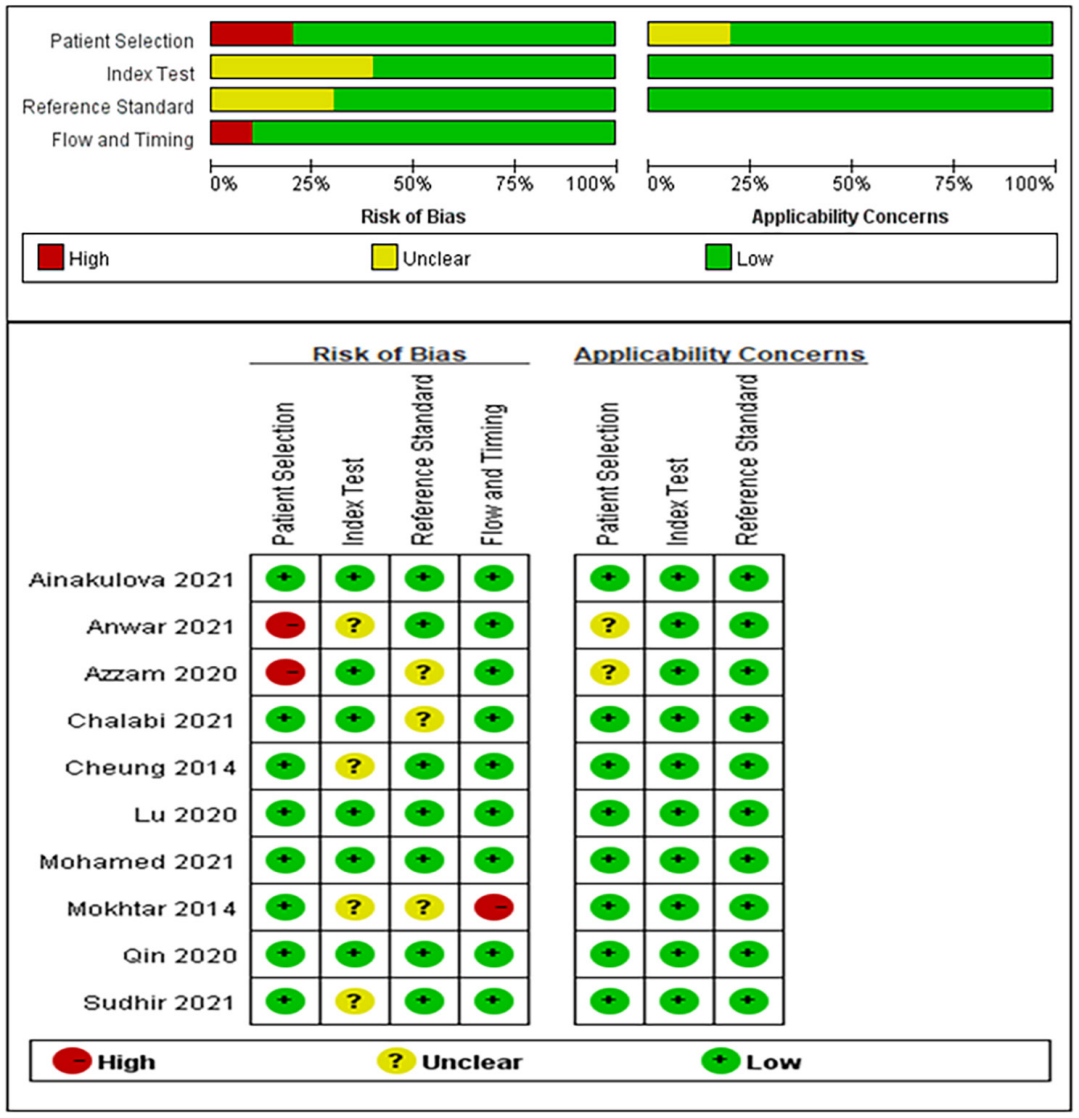
Quality evaluation of studies included in the meta‐analysis.

A total of 827 patients with 958 lesions, ranging in age from 15 to 78 years, were included. Among the included studies, three^11^, ^15^, ^19^ were retrospective studies, and the remaining seven^12^, ^13^, ^14^, ^16^, ^17^, ^18^, ^20^ were prospective studies. Table 1 and Additional Files S2 and S3 show the characteristics of the included studies. The details of the risk of bias and clinical applicability of each study are shown in Figure 2. Three studies^12^, ^13^, ^18^ were at a high risk of bias, and the remaining seven studies were at a low risk. All the studies had high clinical applicability.

#### 3.2.1 Diagnostic performance of CEM

**FIGURE 3:**
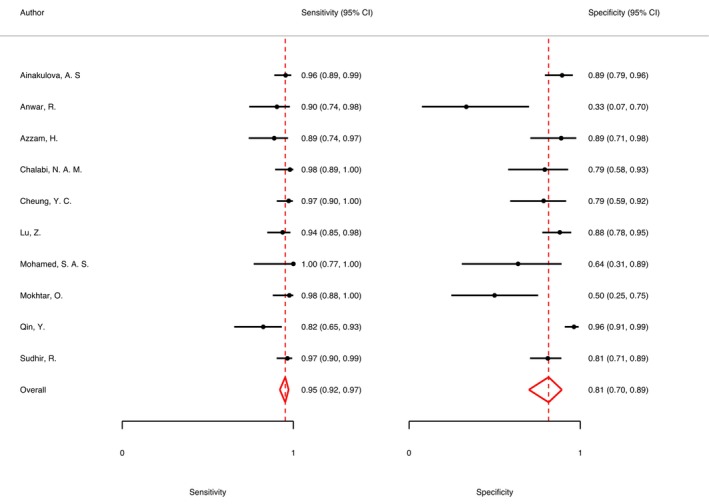
Forest plot of contrast‐enhanced spectral imaging (CEM) sensitivity and specificity.

**FIGURE 4:**
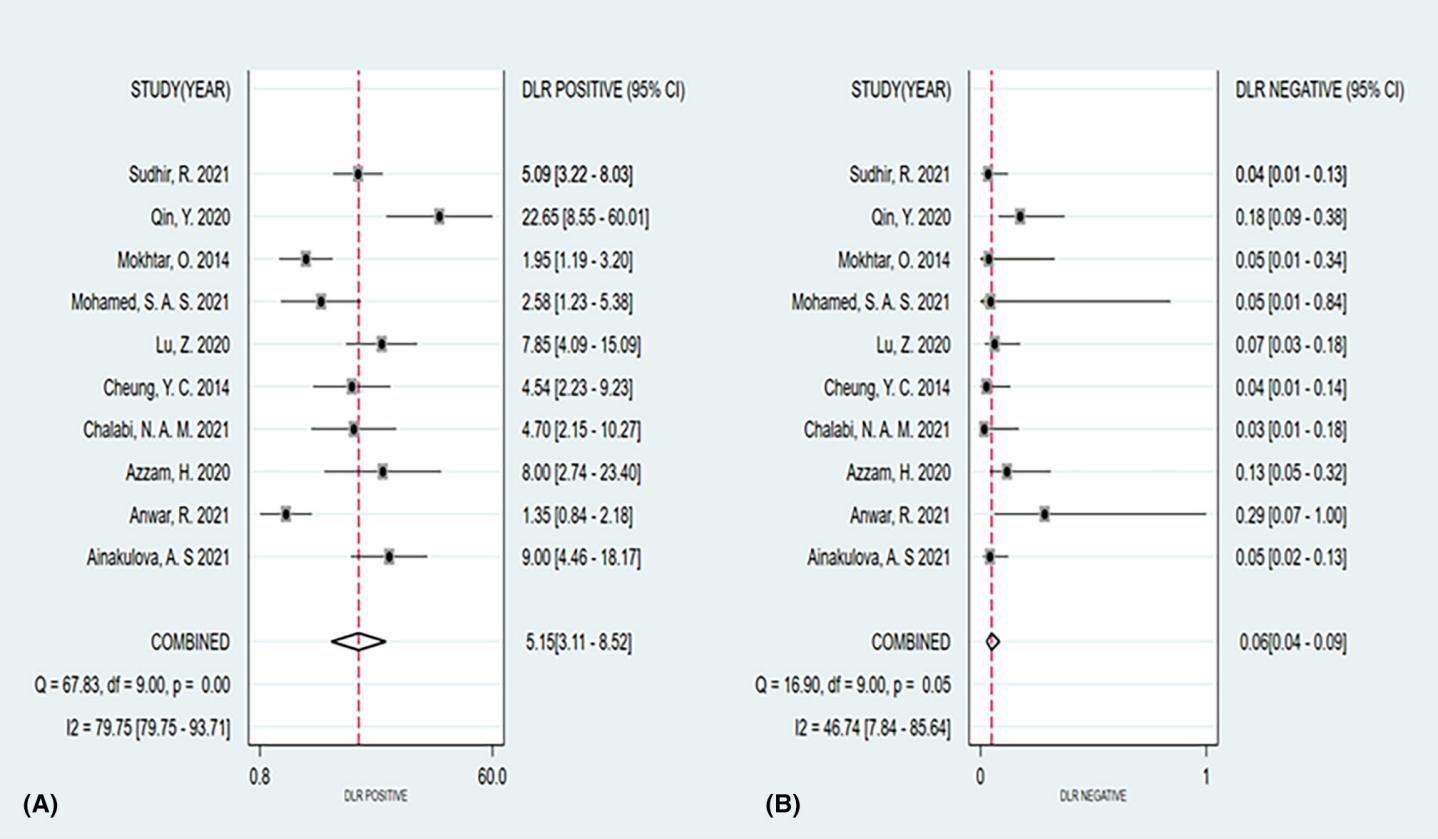
Forest plot of contrast‐enhanced spectral imaging (CEM) positive likelihood ratio (PLR) (A) and negative likelihood ratio (NLR) (B).

**FIGURE 5:**
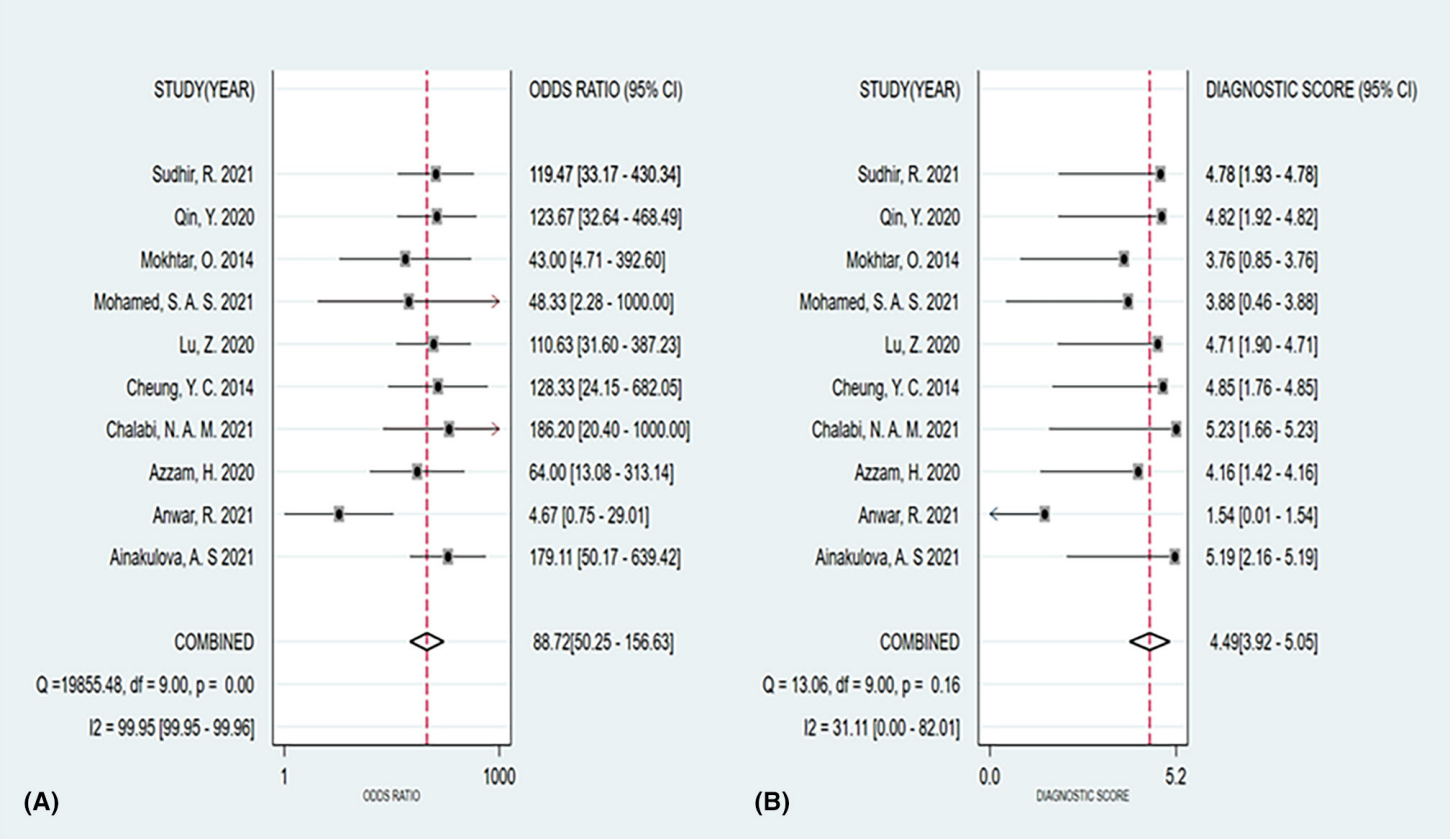
Forest plot of contrast‐enhanced spectral imaging (CEM) diagnostic odds ratio DOR (A) and the diagnostic score (B).

**FIGURE 6:**
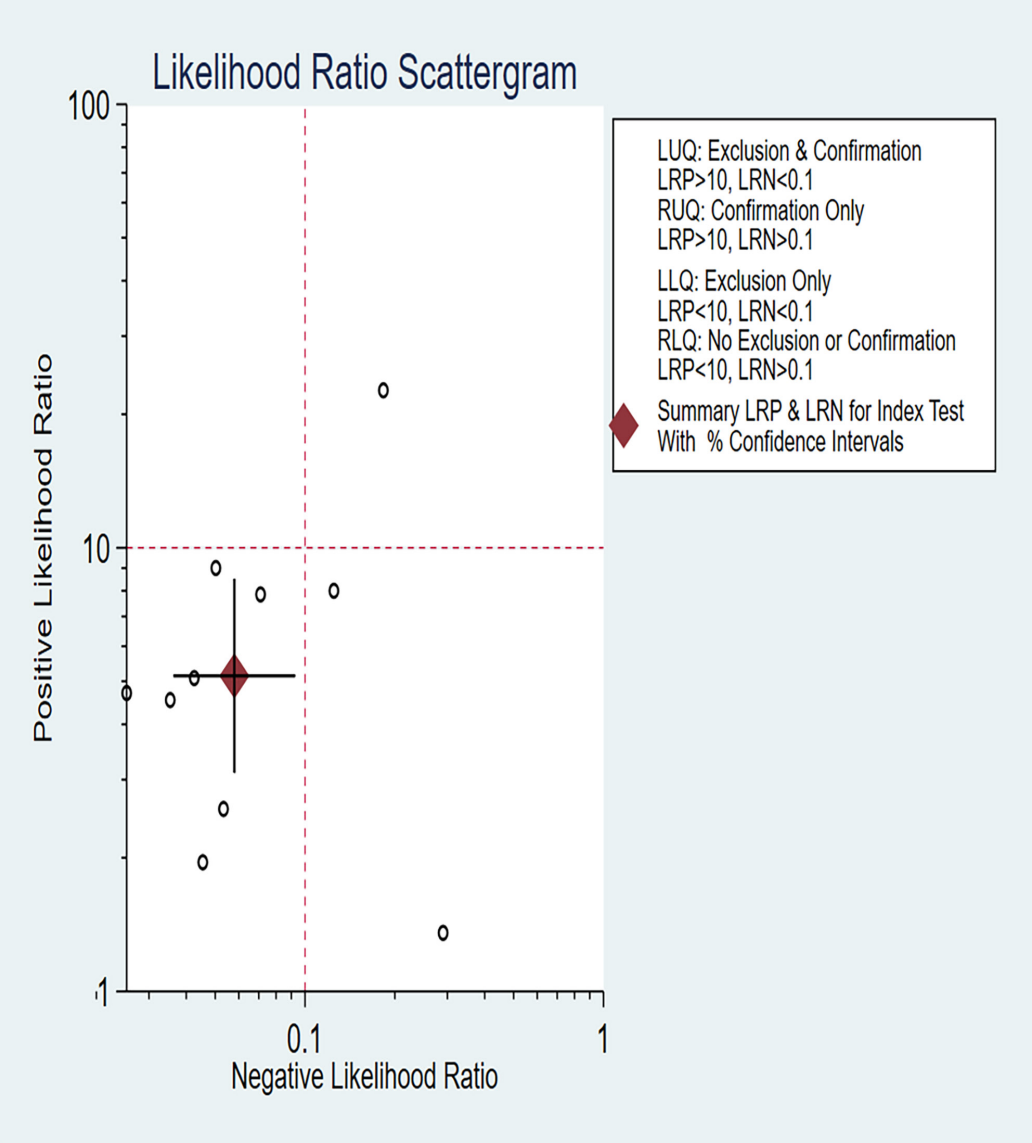
Point plot of positive likelihood ratio (PLR) and negative likelihood ratio (NLR).

**FIGURE 7:**
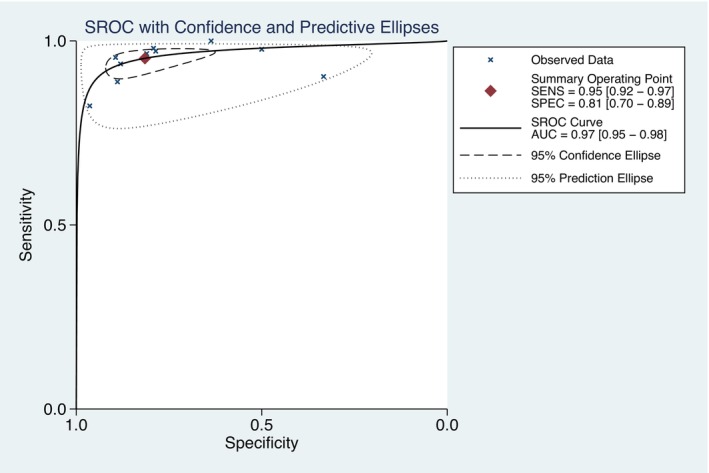
SROC curves for contrast‐enhanced spectral imaging (CEM).

The summary sensitivity, specificity, PLR, and NLR of CEM for the diagnosis of suspicious lesions in dense breasts were 0.95 (95% CI, 0.92–0.97), 0.81 (95% CI, 0.70–0.89), 5.15 (95% CI, 3.11–8.52), and 0.06 (95% CI, 0.04–0.09), respectively. The summary DOR was 88.72 (95% CI, 50.25–156.63), and the diagnostic score was 4.49 (95% CI, 3.92–5.05; Figures 3, 4, 5, 6). The SROC curve is shown in Figure 7 with an AUC of 0.97 (95% CI, 0.95–0.98).

**FIGURE 8:**
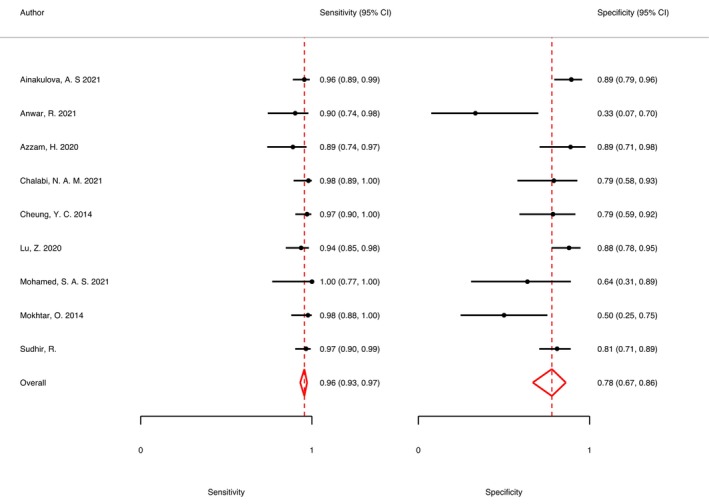
Sensitivity analysis, after excluding Qin et al.'s^19^ study, forest plot of the remaining nine studies.

**FIGURE 9:**
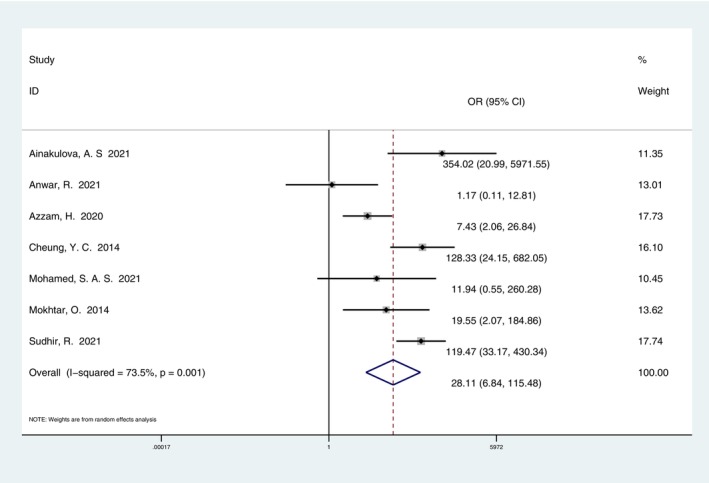
Forest plot of enhanced lesion relative malignancy. The forest map shows that the proportion of malignant lesions was higher than that of benign when the lesions were enhanced. Weights are from random effect analysis.

**FIGURE 10:**
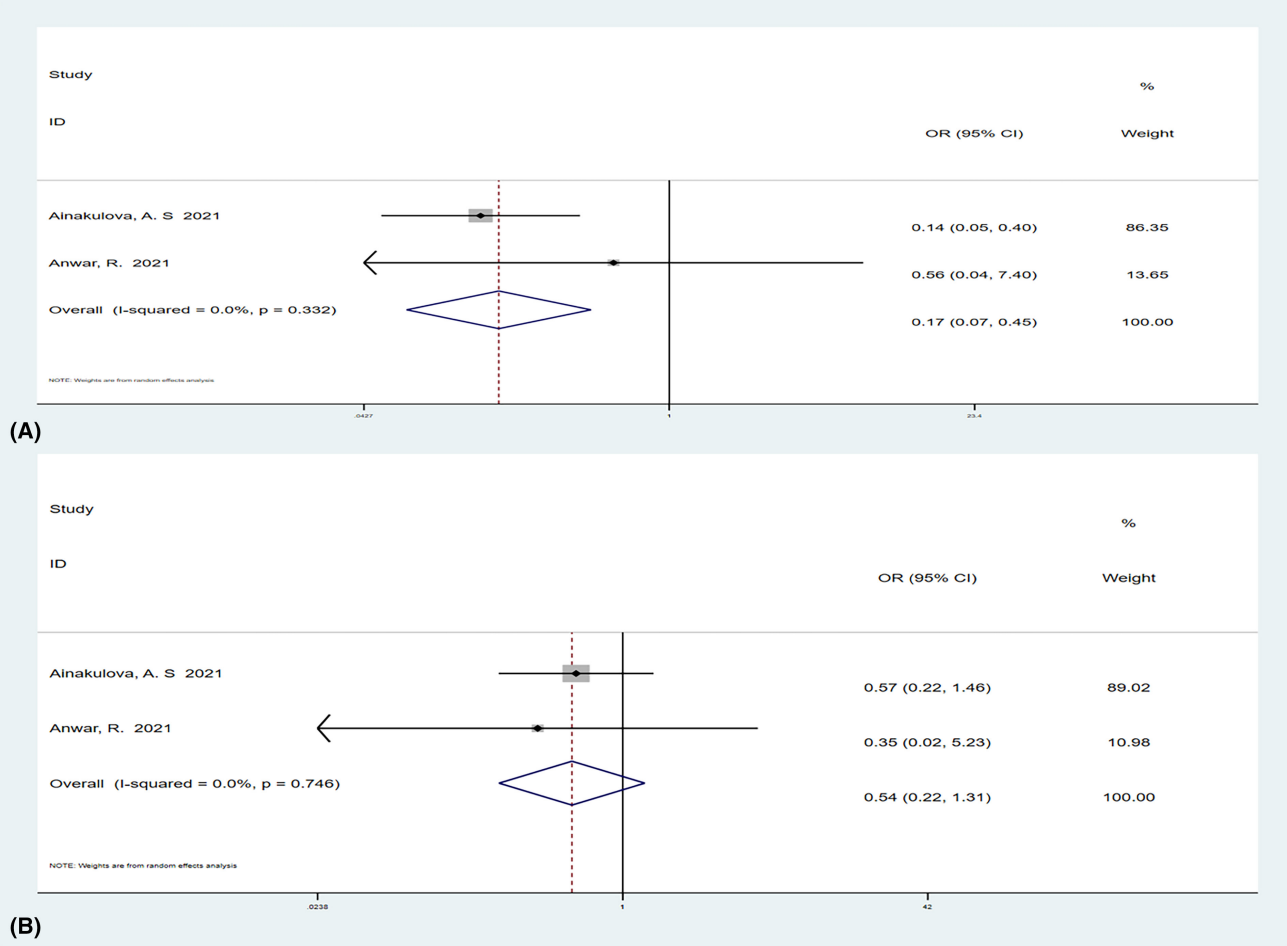
Forest plot of heterogeneous enhancement (A) and rim enhancement (B) relative malignancy. Weights are from random effect analysis.

**FIGURE 11:**
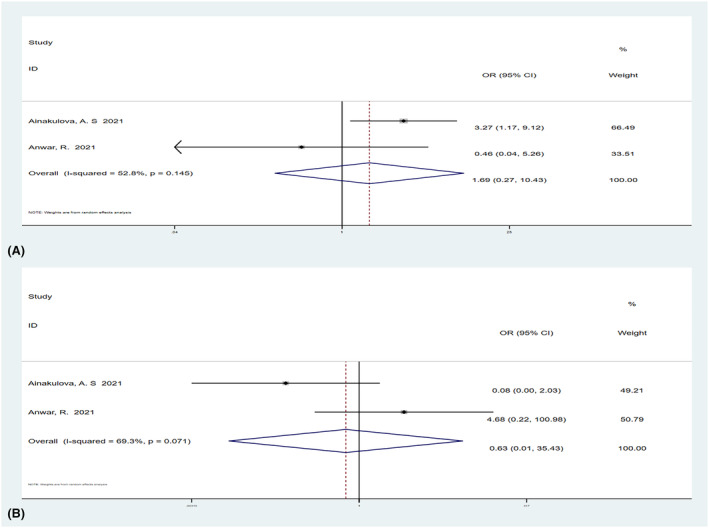
Forest plot of circumscribed margins (A) and homogeneous enhancement (B) relative malignancy. Weights are from random effect analysis.

#### 3.2.2 Heterogeneity

There was heterogeneity in the sensitivity (bivariate *I* ^2^ = 31.56%) and specificity (bivariate *I* ^2^ = 67.66%) of the included studies. The causes of heterogeneity in the diagnostic studies were divided into threshold and non‐threshold effects. As observed from the distribution of non‐“shoulder arm” points on the SROC curve plane, the threshold effects were not the cause of this heterogeneity. Furthermore, the non‐threshold effects were analyzed using a meta‐regression. The meta‐regression was used to assess the following aspects of the 10 studies (Table 2): the study design (prospective or retrospective), sample size (>50 or <50), image acquisition (cranio‐caudal [CC] view first or mediolateral oblique [MLO] view first), study settings (more benign lesions or malignant lesions), and reference standards (only histopathology or mixed reference standards). The study setting had a significant effect on the heterogeneity in sensitivity (*p* = 0.03) and the heterogeneity in specificity (*p* = 0.01), while the sample size had a significant effect on the heterogeneity in specificity (*p* = 0.01; Table 3). In addition, a sensitivity analysis was performed on the included studies. The sensitivity analysis showed that Qin et al.'s study had a significant impact on the heterogeneity. After excluding this study, the remaining nine studies showed no significant heterogeneity in sensitivity (bivariate *I* ^2^ = 2.43%) and a slightly reduced specificity heterogeneity (bivariate *I* ^2^ = 20.19%). The analysis of the remaining nine studies yielded a summary sensitivity of 0.96 (95% CI, 0.93–0.97) and specificity of 0.78 (95% CI, 0.67–0.86; Figure 8).

**TABLE 2:** Characteristics of the 10 included studies.

Study	Prodesign (prospective)	Sample size (>50)	Image acquisition (CC view first)	Setting (proportion of benign and malignant lesions)	Reference standard (histopathology only)
Ainakulova et al.^11^	No	Yes	Yes	<1	Yes
Anwar et al.^12^	Yes	No	No	<1	Yes
Azzam et al.^13^	Yes	No	No	<1	No
Chalabi et al.^14^	Yes	Yes	Yes	<1	No
Cheung et al.^15^	No	Yes	Yes	>1	Yes
Lu et al.^16^	Yes	Yes	Yes	>1	Yes
Mohamed et al.^17^	Yes	No	Yes	<1	Yes
Mokhtar et al.^18^	Yes	Yes	No	<1	No
Qin et al.^19^	No	Yes	Yes	>1	Yes
Sudhir et al.^20^	Yes	Yes	Yes	<1	Yes

**TABLE 3:** Results of the meta‐regression analysis of the 10 studies.

	Estimate (95% CI)	Coef.	*z*	*p*‐Value
Sensitivity				
Prodesign	0.96 (0.92–0.98)	3.12	0.81	0.42
Sample size	0.94 (0.90–0.97)	2.83	−0.41	0.68
Image acquisition	0.96 (0.92–0.98)	3.08	0.60	0.55
Setting (proportion of benign and malignant lesions)	0.89 (0.80–0.95)	2.13	−2.24	0.03
Reference standard	0.95 (0.91–0.97)	2.95	−0.26	0.80
Specificity				
Prodesign	0.76 (0.62–0.86)	1.13	−1.95	0.05
Sample size	0.89 (0.80–0.94)	2.66	2.55	0.01
Image acquisition	0.86 (0.76–0.92)	1.79	1.97	0.05
Setting (proportion of benign and malignant lesions)	0.93 (0.83–0.98)	2.66	2.38	0.02
Reference standard	0.83 (0.71–0.91)	1.16	0.64	0.52

Abbreviations: CI, confidence interval; Coef, coefficient.

### 3.3 CEM findings in benign and malignant lesions

Seven studies^11^, ^12^, ^13^, ^15^, ^17^, ^18^, ^20^ reported the CEM findings of benign and malignant lesions. In the analysis of 609 lesions, the relative malignancy OR value of the enhanced lesions was 28.11 (95% CI, 6.84–115.48). Enhanced lesions and malignancy were statistically correlated (Figure 9).

Two studies^11^, ^12^ with a total of 195 lesions examined both the benign and malignant lesions for margins (circumscribed or non‐circumscribed) and internal enhancement (homogeneous, heterogeneous, or rim enhancement). The results showed that the OR value of circumscribed margins relative to malignancy was 0.17 (95% CI, 0.07–0.45), and circumscribed margin was statistically associated with malignant outcomes (Figure 10A). The OR value of homogeneous enhancement relative to malignant lesions was 0.54 (95% CI, 0.22–1.33; Figure 10B). The OR value of heterogeneous enhancement relative to malignancy was 1.69 (95% CI, 0.27–10.43; Figure 11A), and the OR value of rim enhancement relative to malignancy was 0.63 (95% CI, 0.01–35.43; Figure 11B). Homogeneous, heterogeneous, and rim enhancement were not statistically associated with malignancy.

## 4 DISCUSSION

This systematic review and meta‐analysis assessed 10 articles that included 827 patients and 958 lesions. The results of the analysis of the included studies indicated that CEM has good performance for the diagnosis of suspicious lesions in dense breasts.

High breast density is a common risk factor for breast cancer. Dense breasts have a 2.92–4.24 times higher risk of breast cancer than fatty breasts.^21^, ^22^ Not only do high‐density breasts have a low contrast with lesions, but the superposition of dense breast tissue reduces the sensitivity of mammographic diagnosis significantly.^23^ Multiple studies have shown that the sensitivity of mammography to breast cancer decreases with increasing breast density.^24^, ^25^, ^26^, ^27^ At present, the diagnostic performance of CEM in dense breasts has only been evaluated as a sub‐analysis in the systematic review of Cozzi et al.^28^

The QUADAS‐2 quality assessment of the included studies showed good clinical applicability and a low risk of bias. Only three articles were at a high risk of bias in terms of patient selection, flow, and timing. In terms of patient selection, two studies^12^, ^13^ did not clarify whether the patients were selected using randomization or continuous enrollment. In terms of flow and timing, one study^18^ did not use the same reference standards as the other studies, mainly because of the lack of follow‐up imaging in patients with benign lesions.

The results of this meta‐analysis showed that CEM had high sensitivity and specificity for the diagnosis of breast cancer in dense breasts (0.95 and 0.81, respectively). Our results are consistent with the findings of Cozzi et al.^28^ who reported that CEM has a diagnostic sensitivity of 95% and a specificity of 78% for lesions in dense breasts. In this meta‐analysis, CEM PLR was 5.15.This may have been due to the widely varying percentage (6%–28%) of precancerous or benign lesions, such as fibroadenomas, intraductal papilloma, and fibrosclerosis, showing an increased iodine uptake.^24^, ^29^ This also reminds us to be alert to the possibility of false‐positives when the CEM indicates the presence of positive lesions. The NLR was 0.06, indicating that breast cancer can be excluded when the CEM indicates the presence of negative lesions. These results suggested that CEM has a good diagnostic performance in dense breasts, especially in the case of nonnegative examination results, which may be beneficial in reducing the number of unnecessary biopsies.

The meta‐analysis of the sensitivity and specificity of the included studies revealed significant heterogeneity. We assessed the causes of heterogeneity using threshold and non‐threshold effects. According to the SROC curve plane, the distribution of all the studies did not match the “shoulder arm” point distribution, so the included studies did not have a threshold effect. The meta‐regression analysis showed that the heterogeneity in the sensitivity was related to the study setting, and that the heterogeneity in the specificity was related to the study setting and the sample size. The sensitivity analysis using the article‐by‐article exclusion method found that the heterogeneity of sensitivity was reduced significantly after the study by Qin et al. was excluded.^19^ Therefore, this study may have significantly contributed to heterogeneity, especially because of the skewed proportion of benign and malignant lesions. Compared with other studies, the number of benign lesions in this study was 3.24 times higher than that of malignant lesions. In other studies, the number of benign lesions was less or similar to that of malignant lesions. The combined sensitivity and specificity of the remaining studies except for this study were 0.96 and 0.78, respectively.

This meta‐analysis considered CEM characteristics of 609 benign and malignant lesions and found that the relative malignancy OR value of the enhanced lesions was greater than one, suggesting that the enhanced lesions were more likely to be malignant than benign. This was consistent with current research reports.^30^, ^31^, ^32^

This meta‐analysis analyzed the association of margins and internal enhancement with malignancy in 195 lesions and found that the OR value of circumscribed margins relative to malignancy was 0.17 (95% CI, 0.07–0.45) and that circumscribed margin was statistically associated with malignancy. When a lesion has circumscribed margins, its probability of being malignant is lower than that of being benign. This was consistent with a recent report.^33^ In this systematic review and meta‐analysis, homogeneous, heterogeneous, and rim enhancement were not statistically associated with malignancy. This was inconsistent with the findings of previous studies^11^, ^33^ which indicated that malignant lesions showed a heterogeneous enhancement while benign lesions showed a homogeneous enhancement. Due to the small sample size, the results may be contradictory and need to be carefully evaluated. Moreover, the description of enhanced lesions was obtained mainly using subjective observations. It is recommended that quantitative studies be conducted in the future, to explore the correlation between the lesions and enhanced performance, and to try and rule out the differences that may be due to subjectivity in diagnosis.

Our study had certain limitations. First, the studies included have extremely heterogeneous populations, where CEM is executed in extremely different diagnostic workup settings (symptomatic patients, postoperative, etc.).Second, this systematic review focused on studies from the second‐level diagnostic setting (i.e., the diagnostic workup of suspicious lesions) where the prevalence of cancer is, a priori, obviously much higher than in the screening setting, and that this hinders the generalizability of the results of this meta‐analysis. Third, five of the included studies lacked patient data precisely describing ACR C and D distributions and information on the radiologists involved in image interpretation, which may have been an important reason for the heterogeneity. Lastly, the margins and enhancement findings of benign and malignant lesions were evaluated only in a small number of studies and patients. Third, although heterogeneity of sensitivity decreased when the study by Qin et al. was excluded, the heterogeneity of specificity did not decrease significantly, indicating that further analysis of study heterogeneity is needed.

## 5 CONCLUSION

In conclusion, our systematic review and meta‐analysis found that, despite high heterogeneity, CEM had a high diagnostic value in suspicious lesions in dense breasts. When they were enhanced, irregular, or speculated, the lesions were mostly malignant.

## AUTHOR CONTRIBUTIONS

**Shu‐ting Lin:** Data curation (lead); formal analysis (lead); investigation (lead); methodology (lead); writing – original draft (lead). **Hong‐jiang Li:** Data curation (supporting). **Yi‐zhong Li:** Formal analysis (supporting). **Qian‐qian Chen:** Methodology (supporting). **Jia‐yi Ye:** Validation (supporting). **Shu Lin:** Conceptualization (supporting); writing – review and editing (supporting). **Si‐qing Cai:** Conceptualization (equal); writing – review and editing (equal). **Jian‐guo Sun:** Conceptualization (equal); writing – review and editing (equal).

## CONFLICT OF INTEREST STATEMENT

The authors declare that the research was conducted in the absence of any commercial or financial relationships that could be construed as a potential conflict of interest.

## Supporting information


Data S1:

## Data Availability

The data that support the findings of this study are available on request from the corresponding author, upon reasonable request.
