# Supplementary material for: Diagnostic performance of contrast‐enhanced mammography for suspicious findings in dense breasts: A systematic review and meta‐analysis
Source: Cancer Med. 2024 Apr 25;13(8):e7128. doi: 10.1002/cam4.7128 (PMC11043676; doi:10.1002/cam4.7128)
Supplement: Supplementary file 1 — Data S1: [file CAM4-13-e7128-s001.docx]

Additional file 1

| DATABASE | Mesh words AND Free words | Results |
| --- | --- | --- |
| Pubmed | (((dense[All Fields]) OR (density[All Fields])) AND ((((((((((((((((((((((((((((((((((Mammary Glands, Human[MeSH Terms]) OR (breast[All Fields])) OR (breasts[All Fields])) OR (Gland, Human Mammary[All Fields])) OR (Glands, Human Mammary[All Fields])) OR (Mammary Gland, Human[All Fields])) OR (Mammary Glands[All Fields])) OR (Gland, Mammary[All Fields])) OR (Glands, Mammary[All Fields])) OR (Human Mammary Gland[All Fields])) OR (Human Mammary Glands[All Fields])) OR (Mammary Gland[All Fields])) OR (Mammary Lobules, Human[All Fields])) OR (Human Mammary Lobule[All Fields])) OR (Human Mammary Lobules[All Fields])) OR (Mammary Lobule, Human[All Fields])) OR (Mammary Ducts, Human[All Fields])) OR (Duct, Human Mammary[All Fields])) OR (Ducts, Human Mammary[All Fields])) OR (Human Mammary Duct[All Fields])) OR (Human Mammary Ducts[All Fields])) OR (Mammary Duct, Human[All Fields])) OR (Mammary Alveoli, Human[All Fields])) OR (Alveoli, Human Mammary[All Fields])) OR (Alveolus, Human Mammary[All Fields])) OR (Human Mammary Alveoli[All Fields])) OR (Human Mammary Alveolus[All Fields])) OR (Mammary Alveolus, Human[All Fields])) OR (Mammary Epithelia, Human[All Fields])) OR (Epithelia, Human Mammary[All Fields])) OR (Human Mammary Epithelia[All Fields])) OR (Mammary Epithelium, Human[All Fields])) OR (Epithelium, Human Mammary[All Fields])) OR (Human Mammary Epithelium[All Fields]))) AND ((((contrast-enhanced spectrum mammography[All Fields]) OR (spectral contrast-enhanced mammography[All Fields])) OR (contrast enhancement spectrum mammogram[All Fields])) OR (contrast enhanced digital mammography[All Fields])) OR (contrast-enhanced mammography[All Fields])) | 345 |
| Embase | ('dense' OR 'density') AND ('mammary glands, human' OR 'breast' OR 'breasts' OR 'gland, human mammary' OR 'glands, human mammary' OR 'mammary gland, human' OR 'mammary glands' OR 'gland, mammary' OR 'glands, mammary' OR 'human mammary gland' OR 'human mammary glands' OR 'mammary gland' OR 'mammary lobules, human' OR 'human mammary lobule' OR 'human mammary lobules' OR 'mammary lobule, human' OR 'mammary ducts, human' OR 'duct, human mammary' OR 'ducts, human mammary' OR 'human mammary duct' OR 'human mammary ducts' OR 'mammary duct, human' OR 'mammary alveoli, human' OR 'alveoli, human mammary' OR 'alveolus, human mammary' OR 'human mammary alveoli' OR 'human mammary alveolus' OR 'mammary alveolus, human' OR 'mammary epithelia, human' OR 'epithelia, human mammary' OR 'human mammary epithelia' OR 'mammary epithelium, human' OR 'epithelium, human mammary' OR 'human mammary epithelium') AND ('contrast-enhanced spectrum mammography' OR 'spectral contrast-enhanced mammography' OR 'contrast enhancement spectrum mammogram' OR 'contrast enhanced digital mammography' OR 'contrast-enhanced mammography'  ) | 146 |
| Cochran | (((dense) OR (density)) AND ((((((((((((((((((((((((((((((((((Mammary Glands, Human[MeSH Terms]) OR (breast)) OR (breasts)) OR (Gland, Human Mammary)) OR (Glands, Human Mammary)) OR (Mammary Gland, Human)) OR (Mammary Glands)) OR (Gland, Mammary)) OR (Glands, Mammary)) OR (Human Mammary Gland)) OR (Human Mammary Glands)) OR (Mammary Gland)) OR (Mammary Lobules, Human)) OR (Human Mammary Lobule)) OR (Human Mammary Lobules)) OR (Mammary Lobule, Human)) OR (Mammary Ducts, Human)) OR (Duct, Human Mammary)) OR (Ducts, Human Mammary)) OR (Human Mammary Duct)) OR (Human Mammary Ducts)) OR (Mammary Duct, Human)) OR (Mammary Alveoli, Human)) OR (Alveoli, Human Mammary)) OR (Alveolus, Human Mammary)) OR (Human Mammary Alveoli)) OR (Human Mammary Alveolus)) OR (Mammary Alveolus, Human)) OR (Mammary Epithelia, Human)) OR (Epithelia, Human Mammary)) OR (Human Mammary Epithelia)) OR (Mammary Epithelium, Human)) OR (Epithelium, Human Mammary)) OR ((Human Mammary Epithelium))) AND ((((contrast-enhanced spectrum mammography) OR (spectral contrast-enhanced mammography)) OR (contrast enhancement spectrum mammogram)) OR (contrast enhanced digital mammography)) OR (contrast-enhanced mammography)) | 9 |

Additional file 2

|  | Malignant | | Benign | |
| --- | --- | --- | --- | --- |
|  | enhancement | | enhancement | |
| Author | yes | no | yes | no |
| Ainakulova, A. S | 89 | 0 | 22 | 44 |
| Anwar, R. | 28 | 3 | 8 | 1 |
| Azzam, H. | 32 | 4 | 14 | 13 |
| Cheung, Y. C. | 70 | 2 | 6 | 22 |
| Mohamed, S. A. S. | 14 | 0 | 8 | 3 |
| Mokhtar, O. | 43 | 1 | 11 | 5 |
| Sudhir, R. | 84 | 3 | 15 | 64 |

Additional file 3

|  | Author | Ainakulova, A. S | Anwar, R. |
| --- | --- | --- | --- |
|  | Margins |  |  |
|  | circumscribed | 13 | 3 |
|  | non-circumscribed | 76 | 16 |
| Malignant | internal enhancement |  |  |
|  | homogeneous | 40 | 2 |
|  | heterogeneous | 49 | 11 |
|  | rim | 0 | 6 |
|  | Margins |  |  |
|  | circumscribed | 12 | 1 |
|  | non-circumscribed | 10 | 3 |
| Benign | internal enhancement |  |  |
|  | homogeneous | 13 | 1 |
|  | heterogeneous | 6 | 3 |
|  | rim | 1 | 0 |
